# Advancing Egg Freshness Evaluation with Integrated AI and Spectroscopy

**DOI:** 10.3390/foods15132259

**Published:** 2026-06-23

**Authors:** Ziye Xu, Dachen Wang, Zhihui Zhu, Yushan Jiang, Huang Dai, Yingli Wang, Qiaohua Wang

**Affiliations:** 1 College of Engineering, Huazhong Agricultural University, Wuhan 430070, China; ziye_xu@webmail.hzau.edu.cn (Z.X.); zzh@mail.hzau.edu.cn (Z.Z.); ysjiang0921@126.com (Y.J.); wqh@mail.hzau.edu.cn (Q.W.); 2 College of Mechanical and Electronic Engineering, Nanjing Forestry University, Nanjing 210037, China; dcwang@njfu.edu.cn; 3 College of Food Science and Engineering, Wuhan Polytechnic University, Wuhan 430023, China; huangdai9@126.com

**Keywords:** egg freshness, spectroscopic techniques, multi-sensor fusion, artificial intelligence technology

## Abstract

As hen eggs are a primary source of high-quality dietary protein, egg freshness is fundamentally linked to biochemical alterations during storage, including moisture redistribution, protein degradation, and fluctuating chemical profiles. Accurate assessment of these internal changes is paramount for quality control; nonetheless, conventional analytical techniques remain predominantly destructive, rendering them impractical for high-throughput industrial monitoring. While existing literature has explored individual spectroscopic methods, the synergistic potential of multi-sensor integration and advanced artificial intelligence (AI) algorithms remains insufficiently synthesized. This review systematically evaluates recent breakthroughs in integrating AI with diverse spectroscopic modalities for non-destructive freshness quantification, including Visible-Near-Infrared (VIS-NIR), Raman, Fluorescence, and Hyperspectral Imaging (HSI). We elucidate the underlying mechanisms of spectral response to internal quality degradation and discuss the evolution of data-driven modeling from traditional chemometrics to sophisticated deep learning architectures. Furthermore, this work identifies critical bottlenecks in real-time industrial implementation and proposes future research trajectories toward intelligent multi-sensor fusion platforms.

## 1. Introduction

Hen eggs represent an indispensable source of high-biological-value proteins and essential lipids within the human diet [1]. Their multifaceted functional properties—including gelation, foaming stability, and natural pigmentation—render them fundamental constituents in diverse food formulations [2]. Over the past decade, global egg production has sustained a robust growth trajectory; as of 2024, output reached approximately 1.3 trillion units, with projections estimating a further increase to 90 million tons by 2030 [3]. This industrial expansion necessitates more rigorous storage protocols and quality management. Since egg quality is inherently non-improvable post-oviposition and undergoes progressive biochemical degradation during storage, the precision of freshness evaluation is critical to maintaining economic value and mitigating food safety risks [4].

Freshness is the core indicator for evaluating the internal quality of eggs, exerting a direct impact on their nutritional worth and quality characteristics [5]. Key physical indicators—such as the Haugh unit (HU), albumen height, yolk index, and air cell diameter—as well as the albumen pH, which serves as a primary chemical indicator, undergo regular changes due to biochemical reactions and substance migration within stored eggs. These are widely recognized as objective criteria for assessing freshness [6,7]. Specifically, Haugh Unit is an internationally recognized quantitative metric for egg freshness and albumen quality, calculated from egg weight and albumen height, where a higher HU value represents better albumen viscosity and fresher eggs [8]. Albumen height directly reflects the thick albumen status, while yolk index characterizes yolk roundness. Air cell diameter and albumen pH gradually change during storage, both serving as typical markers of egg deterioration. The detection methods for egg freshness can be classified into three main categories: traditional sensory evaluation, physicochemical methods, and modern non-destructive detection methods [9]. Among these, traditional approaches relying on visual inspection, olfactory detection, and auditory evaluation are limited by subjective bias and low efficiency [10]. Physicochemical methods, on the other hand, are plagued by issues such as one-sided results and destructive effects on samples [11]. In contrast, spectral detection technology has seen rapid growth in the field of egg quality testing due to its non-destructive, rapid, and reagent-free nature [12]. A spectrum is a continuous or discontinuous chart formed by arranging electromagnetic radiation according to wavelength or frequency [13]. When light of different wavelengths is shone onto an egg sample, it produces distinct optical responses. Technologies such as Near-Infrared Spectroscopy (NIR), Hyperspectral Imaging (HSI), Raman Spectroscopy (RS), and Fluorescence Spectroscopy (FS) have been developed based on these optical response principles. They have demonstrated significant application potential in egg quality grading and freshness assessment [14]. The present review focuses on the four spectral analysis technologies, systematically summarizes their research progress in the non-destructive detection of the internal quality of fresh eggs, and explores the enabling mechanisms and application value of multi-sensor fusion technology and artificial intelligence (AI) for spectral technology. This paper aims to clarify theoretical research directions for scholars in related fields, establish a systematic application framework, and provide practical guidance for quality control systems in the egg industry.

## 2. Spectroscopic Techniques

### 2.1. Visible-near-Infrared Spectroscopy

The detection principle of visible-near-infrared spectroscopy (VIS-NIRS) is predicated on the characteristic responses of the internal components of eggs to light of varying wavelengths. The visible light band (400–780 nm) is primarily responsible for reflecting information regarding pigments associated with electronic transitions and the appearance of color. The near-infrared band (780–2500 nm) has been shown to coincide with the overtone and combination tone vibrational absorption of hydrogen-containing groups in the primary components of eggs [15].

**Figure 1:**
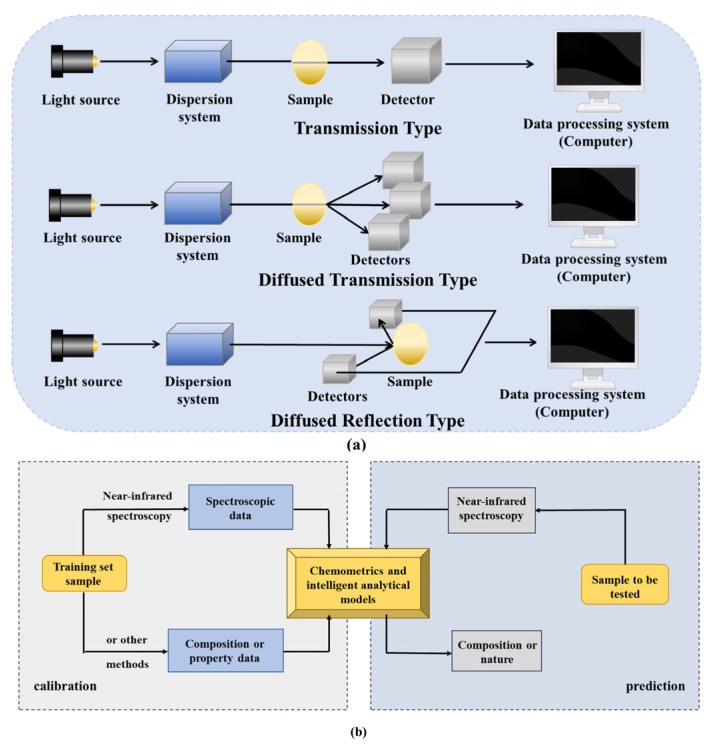
Components of a near-infrared spectroscopy system and the analysis process: (**a**) The basic components of visible-near-infrared spectroscopy (VIS-NIRS). (**b**) Data analysis workflow of near-infrared spectroscopy. Note: The arrow extending from “detection” in Figure 1a and all arrows in Figure 1b indicate the direction of information flow; the remaining arrows indicate the direction of light propagation.

VIS-NIRS measurement methods can be categorized into transmission methods (including direct transmission and diffuse transmission) and reflection methods, which employ different optical path configurations [16]. The specific composition of the system is shown in Figure 1a. Direct transmission requires a 180-degree direct light path and is effective only for thin samples [17]; diffuse transmission and diffuse reflection are more commonly used in food testing. It is evident that the accuracy of near-infrared spectroscopy is susceptible to the influence of eggshell color and surface contaminants. A comparative experiment demonstrated that, because the reflection mode is susceptible to defects on the eggshell surface, the transmission mode provides more reliable information about the internal composition of the egg [18]. For spectra obtained using the diffuse transmission mode, the model can be applied across different egg varieties after applying preprocessing and characteristic wavelength filtering methods to mitigate interference caused by variations in eggshell surfaces [19]. However, the hardware structure for diffuse transmission is more complex and is better suited for high-precision laboratory research, whereas portable industrial devices typically use the diffuse reflection mode [20].

VIS-NIRS analysis technology infers the composition or properties of a sample in a non-destructive manner by collecting the sample’s optical signals. The specific process is illustrated in Figure 1b. First, during the calibration phase, the spectra of a set of calibration samples with known compositions or properties must be measured. These spectra are then combined with reference values to establish a quantitative relationship model between them through chemometrics and intelligent analytical models. In the prediction stage, only the spectrum of the sample needs to be collected and input into this model to quickly obtain the predicted values of its composition or properties. Based on this principle, VIS-NIRS technology can detect changes in the internal chemical composition and physical properties of eggs, thereby realizing the dynamic evaluation of egg freshness.

The evaluation of egg freshness usually relies on key indicators such as Haugh Unit, albumen height, and yolk index [21]. Since eggshells are covered with tiny pores, the internal moisture and carbon dioxide of eggs are exchanged through these pore canals during storage, triggering a series of physical and chemical changes [22]. Studies have demonstrated that storage temperature and duration have a substantial impact on all egg quality indices. Among these indices, egg weight loss, albumen pH, and Haugh unit exhibit the greatest sensitivity to storage conditions [23]. NIRS can sensitively capture these compositional changes; however, its broad and overlapping signals make direct extraction of features corresponding to individual indicators challenging. Therefore, in practical applications, both quantitative and qualitative VIS-NIR analyses require chemometric methods [24].

Chemometric methods and machine learning (ML) models constitute the foundation of AI-enabled spectral modeling and can be classified into unsupervised and supervised learning categories [25]. Near-infrared spectral data are difficult to analyze directly. Unsupervised methods such as Principal Component Analysis (PCA) are often employed for data dimensionality reduction and anomaly detection to simplify the data structure [26]. Quantitative prediction or classification of freshness indicators requires the use of supervised machine learning techniques. Given its efficacy in eliminating the impact of multicollinearity among spectral intervals, Partial Least Squares Regression (PLSR) has become a prevalent linear machine learning method for developing prediction models for continuous indicators such as Haugh units [27]. Support Vector Regression (SVR), on the other hand, demonstrates a marked aptitude for managing complex nonlinear relationships and represents a typical nonlinear AI modeling tool [28]. The combination of auxiliary technologies to optimize wavelength selection and the adoption of different preprocessing methods have become important strategies to improve the accuracy of AI prediction models [29]. In classification applications, due to potential overlap or clustering of sample data in mathematical space, simple differentiation becomes difficult. Machine learning classifiers, including support vector machine classification (SVC) and partial least squares discriminant analysis (PLS-DA), construct linear decision boundaries to achieve an 80% accuracy in freshness classification [30].

After the prediction accuracy of the AI-assisted model meets the required standards, the bottleneck of technological development has shifted toward practical applications. Given that large-scale laboratory instruments are unsuitable for on-site or online detection, the development of portable devices integrated with lightweight machine learning models has become a key innovative direction. Research has confirmed that using a handheld near-infrared spectrometer to detect eggs does not reduce classification accuracy, and the recognition rate for eggs stored at room temperature and in the refrigerator both exceeded 95% [31]. Based on samples collected from commercial egg farms, Cruz-Tirado et al. constructed a simulated online testing system and demonstrated that low-cost portable near-infrared devices are fully competent for the accuracy criteria of on-line monitoring in egg production lines [32]. A portable near-infrared instrument can also effectively monitor egg quality changes under different feeding methods and storage temperatures [33]. In addition, the miniaturized spectrometers with a tunable van der Waals junction reported by Yoon et al. provide a new technical path for the development of portable, AI-embedded sensing devices [34].

To summarize, near-infrared spectroscopy technology has established a complete technical system combined with chemometrics and machine learning in the field of egg freshness detection. A freshness discrimination model based on a fusion of spectral and textural features addressed the shortcomings of single-class features in terms of representational capacity and achieved an accuracy of 92.42%, which is 3.03 percentage points higher than that of single-feature models [35]. This points to a promising direction that future research can focus on the in-depth integration of multi-model fusion, advanced feature extraction algorithms, and portable intelligent devices to enhance detection robustness and accelerate the large-scale application of VIS-NIR-AI integrated systems in the egg industry.

### 2.2. Hyperspectral Imaging Technique

**Figure 2:**
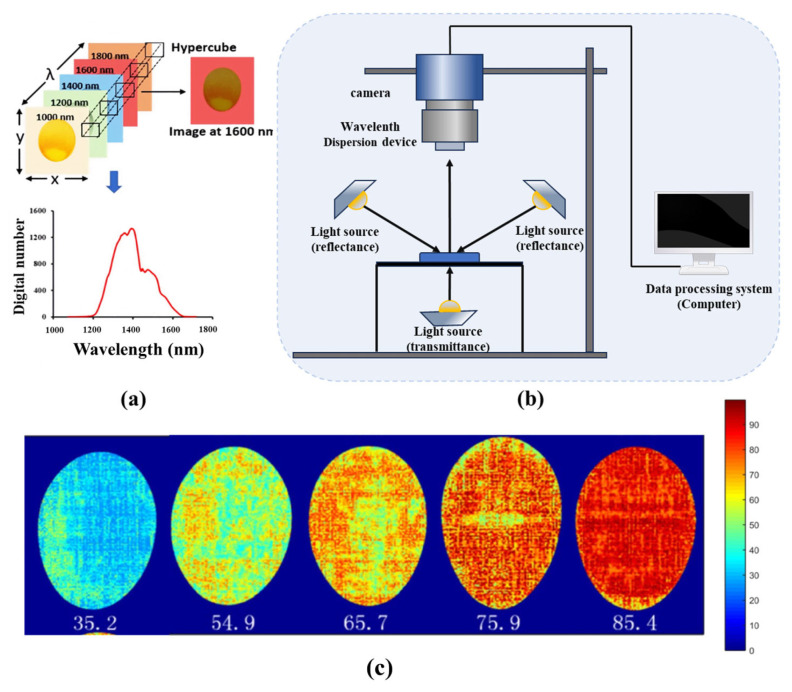
Hyperspectral Imaging (HSI) principle, system components, and sample spatial distribution: (**a**) Schematic representation of the HSI principle [41]. This figure is reused under the CC-BY open access license. (**b**) The basic components of hyperspectral imaging systems. (**c**) Visualization of S-ovalbumin contents in chicken eggs in the chemical map [40]. Note: All arrows in Figure 2b indicate the direction of light propagation.

Hyperspectral Imaging (HSI) combines the spatial imaging capability of computer vision with the compositional analysis power of spectroscopy. By acquiring both spatial images and continuous spectral information across numerous narrow bands, HSI enables qualitative, quantitative, and spatial localization of target analytes through advanced data processing [36]. A typical HSI system is illustrated in Figure 2b. The spectroscopic module, usually equipped with prisms or gratings, performs wavelength separation, while the detector converts optical signals into electrical outputs to synchronously capture spectral and spatial information. All post-processing and analytical operations are implemented in a computer system, covering the full pipeline of data processing and modeling.

In contrast to near-infrared spectroscopy, which captures only point-based spectra, HSI simultaneously records two-dimensional spatial images and full spectral curves for every pixel, forming a three-dimensional spatial–spectral data cube [37]. Figure 2a shows the principle of HSI. It is apparent that HSI enables pixel-level global detection, whereas traditional near-infrared spectroscopy yields only averaged spectral data at a single point, which may mask local anomalies [38]. Studies have confirmed that S-ovalbumin, an indicator of freshness, exhibits a non-uniform distribution during storage [39]. HSI visualizations can intuitively display freshness levels, as shown in Figure 2c, pixels with lower S-ovalbumin content are displayed in blue, indicating greater freshness. Conversely, those with higher content are displayed in red [40].

Benefiting from its high-dimensional information, detection precision, and operational flexibility, HSI has been widely adopted for quality evaluation in various foods, including fruits and vegetables [42], meat [43], aquatic products [44], and cereals [45]. In egg freshness assessment, numerous studies have validated the efficacy of HSI under different imaging modes. Near-infrared reflectance HSI enables visual differentiation of egg freshness through image coloration and has been established as a non-destructive, rapid technique for online grading [39]. Based on transmissive hyperspectral data, a study employed a binary competitive adaptive weighted sampling (BCARS) algorithm to screen for characteristic wavelengths, constructing a model using fewer, more representative feature bands, thereby improving the generalization performance. The corresponding correlation coefficient reached 0.914 [46]. Notably, Dai et al. applied stacking ensemble learning with Digital-to-Analog Converter (DAC), K-Nearest Neighbors (KNN), and Random Forest (RF) as first-layer base learners and DAC as the second-layer meta-learner. By combining the discriminative strengths of multiple individual models to compensate for the misclassification errors inherent in a single classifier, and through collaborative AI modeling, they improved their classification accuracy from 96.25% to 100%. Their work also confirmed the superiority of scattering HSI for egg freshness assessment and revealed the influence of incident angle on detection performance [47].

Although conventional machine learning and ensemble learning have enhanced freshness prediction, these methods remain dependent on handcrafted spectral features. To further strengthen the end-to-end analytical capacity of AI-assisted models for HSI data, recent studies have introduced deep convolutional neural networks (DC-CNN) for non-destructive freshness evaluation, achieving a correlation coefficient of 0.9056 and root-mean-square error of 4.4152, supporting reliable HU prediction [48]. Additionally, the innovative Hyperspectral Egg Freshness Inspection Technology (HEFIT) and hyperspectral egg defect inspection technique (HEDIT), which approach the problem from the perspectives of factory production lines and consumers, respectively, achieve 99% accuracy by combining wavelength selection with deep neural networks and convolutional neural networks, marking a breakthrough in real-time industrial inspection [49].

Although HSI has demonstrated excellent performance in laboratory and pilot tests, it still faces critical limitations in practical deployment, including high instrumental cost, hardware constraints, ultra-high data dimensionality, and computational inefficiency [50]. As a result, it has not yet been adopted for large-scale commercial use in the industry. Recent investigations have demonstrated that these bottlenecks can be alleviated using hyperspectral image reconstruction techniques based on advanced deep learning (DL) frameworks such as dense hyperspectral convolutional neural networks (HSCNN-D), which offer considerable potential for accelerating processing efficiency [51]. With the support of high-speed communication, state-of-the-art machine learning, and hyperspectral reconstruction technologies, the translation and large-scale application of HSI in the egg industry are expected to advance rapidly [41].

### 2.3. Raman Spectroscopy

**Figure 3:**
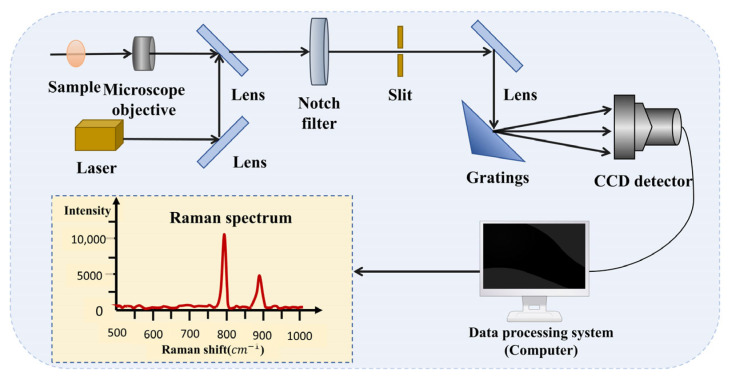
Composition of Raman spectroscopy: Composition of the Raman spectroscopy system. Note: Except for the arrows extending from the computer, which indicate the direction of data flow, all other arrows indicate the direction of light propagation.

Raman spectroscopy is a molecular vibrational spectroscopic technique based on the inelastic light scattering phenomenon known as the Raman effect [52]. Its detection system consists primarily of three components: an excitation source, a wavelength-separating device, and a detector [53], as illustrated in Figure 3. Accumulating evidence demonstrates that Raman spectroscopy holds substantial promise for egg quality and safety assessment [54]. During the testing process, this technology eliminates the need for sample pretreatment such as crushing or dissolution, thereby preserving the integrity and original properties of the eggs while reducing costs. Regarding detection performance, this technology leverages its high signal-to-noise ratio and exceptional stability to precisely identify molecular vibration changes within eggs. It rapidly acquires molecular structure and chemical composition data, providing critical support for structural characterization of components such as proteins, carbohydrates, and lipids [55].

In practical egg quality testing applications, researchers have progressively expanded the analytical scope of Raman spectroscopy. Through systematic comparative experiments, Liu et al. identified the optimal sampling site on intact eggs for Raman spectral acquisition and developed a PLSR model that achieved correlation coefficients exceeding 0.9 for the prediction of critical egg quality parameters, including Haugh unit, albumen pH, and air cell diameter [56]. Furthermore, a growing body of research has explored the application potential of multi-sensor fusion-based analytical platforms that integrate Raman spectroscopy with complementary analytical techniques. For instance, the combination of Raman spectroscopy with near-infrared spectroscopy and nuclear magnetic resonance (NMR) spectroscopy captures more comprehensive molecular information and enhances the predictive accuracy of chemometric models; this multi-sensor fusion strategy has already been successfully implemented for quality assessment of muscle foods, including fish and red meat [57].

Beyond conventional egg quality metrics (e.g., Haugh unit and yolk index), Raman spectroscopy enables the identification of molecular biomarkers strongly associated with egg quality, most notably carotenoids. Carotenoids are crucial in Raman measurements due to their strong resonance-enhanced signals and unique characteristic peaks [58]. However, conventional Raman spectroscopy has an inherent weakness of low scattering intensity, which limits its application in the detection of trace components. This fundamental signal weakness is ubiquitous in food industrial scenarios, as only 10^6^–10^8^ incident photons produce effective Raman scattering, and endogenous fluorescent substances in egg samples further suppress valid spectral signals [59]. Accordingly, enhanced Raman spectroscopic techniques have emerged as a prominent research focus in the field. Davari et al. employed micro-Raman spectroscopy to demonstrate a statistically significant correlation between characteristic Raman peaks and egg freshness index. The study further validated that carotenoids, lipids, and proteins can serve as robust Raman biomarkers for egg quality evaluation and confirmed that the characteristic Raman peak intensity of calcium carbonate in eggshells decreases with extended storage time [60]. These findings provide critical experimental support for the development of non-destructive egg freshness detection technologies and offer a valuable methodological reference for the application of enhanced Raman techniques in egg quality assessment.

With its inherent advantages of rapid detection, minimal sample perturbation, non-destructiveness, and high analytical sensitivity, Raman spectroscopy is well-positioned to meet the requirements of efficiency and precision in egg quality testing. However, the heterogeneity of whole eggs, combined with the fixed positioning of sensors on production lines, can easily lead to unstable optical paths and poor sampling representativeness, which severely affects the reproducibility of online inspection results. At the same time, the high cost of the equipment limits its use to the laboratory testing stage [61]. Sampling speed may also be a limiting factor in real-time analysis applications on production lines [62]. As enhancement technologies mature and equipment costs decrease, the prospects for applying this technology in the end-to-end control of egg quality will become even broader [63]. Meanwhile, issues related to the signal-to-noise ratio and complexity of samples necessitate continued innovation in AI-assisted technologies, such as machine learning [64]. Future research should focus on developing efficient anti-interference algorithms to mitigate environmental and sample background interference, as well as on developing miniature detection devices suitable for on-site industrial applications.

### 2.4. Fluorescence Spectroscopy

**Figure 4:**
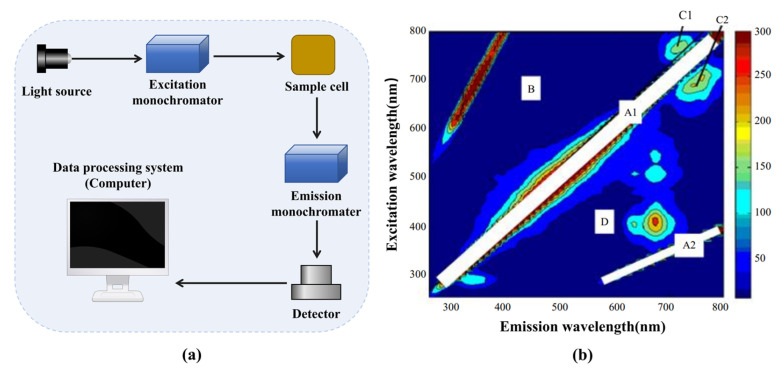
Fluorescence spectral composition and excitation-emission matrix (EEM) spectral characterization of Eggs: (**a**) Composition of fluorescence spectrum. (**b**) EEM spectrum for eggs [74]. Note: In Figure 4b, A1 and A2 are the scattered light interference regions; B is the invalid spectral region; C1 and C2 are the stray light interference regions; and D represents the valid fluorescence signal region.

Fluorescence spectroscopy refers to the characteristic spectral lines produced when a substance is excited and emits light; the resulting spectrum is highly correlated with the substance’s composition, structure, and concentration, and can be used to perform both qualitative and quantitative analysis simultaneously [65,66]. The components of a typical fluorescence spectrometer are shown in Figure 4a.

With the continuous deepening of understanding of the essence of fluorescence, fluorescence spectroscopy technology has been widely applied in food detection [67,68]. It offers significant advantages in organic compound analysis. Certain organic compounds inherently exhibit fluorescence, such as aromatic amino acids like tryptophan and tyrosine found in proteins. Eggs happen to contain these key fluorophores [69]. Based on this, by exciting specific fluorophores inside eggs and determining their emission spectra, it is possible to investigate the molecular interactions of eggs during different storage stages, thereby realizing the evaluation of their freshness.

Compared with traditional techniques that are time-consuming and somewhat destructive, rapid and high-precision fluorescence spectroscopy has emerged as an efficient alternative for the non-destructive assessment of egg freshness. A series of studies have pioneered the basic verification work in this field. Initially, the research focused on the intrinsic fluorescence of egg white, and clearly confirmed that the fluorescence signals of fluorescent Maillard reaction products can serve as “molecular fingerprints” for distinguishing fresh eggs from stale ones [70]. Subsequently, the team further expanded the applicable matrix of fluorescence spectroscopy from egg white to egg yolk, revealing the prominent advantages of vitamin A fluorescence in egg freshness evaluation [71] and verifying the stability and reliability as an evaluation indicator [72,73].

With the growing demand for practical applications, the limitations of conventional fluorescence spectroscopy have become increasingly prominent, while the emergence of specialized fluorescence spectroscopy techniques has provided new avenues for enhancing detection efficiency and accuracy. Synchronous fluorescence spectroscopy can effectively simplify spectral information and reduce interference. Wu et al. established a two-dimensional panoramic spectrum of the synchronous fluorescence excitation-emission matrix (EEM) for eggs, as shown in Figure 4b. This visualizes the distribution of full-spectrum fluorescence signals in eggs, distinguishes between valid signals and interference signals, and identifies key data regions for subsequent modeling of freshness detection. The multiple linear regression (MLR) freshness prediction model constructed based on this data achieved a coefficient of determination ($R_{P}^{2}$) of 0.8879 in the validation set [74].

Fluorescence spectroscopy holds significant potential for egg quality testing; however, due to limitations in the fluorescence properties of the materials being tested, its application as a standalone technique in non-destructive testing of eggs is limited. In practical industrial settings, relying solely on fluorescence spectroscopy for routine egg freshness testing lacks strong market competitiveness; therefore, fluorescence spectroscopy is mostly adopted as a supplementary method in existing studies [75]. Individual variations among samples, as well as fluctuations in ambient temperature and humidity, can interfere with the intrinsic fluorescence signals of the samples, leading to unstable detection accuracy [76]. The heterogeneity of the samples also significantly limits the scope of its practical application [77]. Nevertheless, its integration with other technologies offers a wider range of applications in quality testing—such as for fish [78] and meat [79]—demonstrating the development potential of combining fluorescence spectroscopy with multi-sensor technology. Future efforts should focus on comprehensive research to drive technological innovation and the miniaturization and intelligent upgrading of detection equipment, thereby supporting the high-quality development of the egg industry [80]. Table 1 summarizes the characteristics of several spectroscopic techniques, including fluorescence spectroscopy, in terms of their application potential.

## 3. Multi-Fusion Sensing Technology

Multi-fusion sensing technology refers to a technique that integrates data or features from multiple types of sensors to obtain more comprehensive, accurate, and reliable information. The complexity and diversity of current application scenarios have significantly increased, making traditional single sensors inadequate for meeting the requirements of comprehensive and precise information acquisition. Multi-sensor fusion technology integrates the technical advantages of different sensors to create complementary synergies, thereby enhancing the overall performance and operational stability of the system.

**Figure 5:**
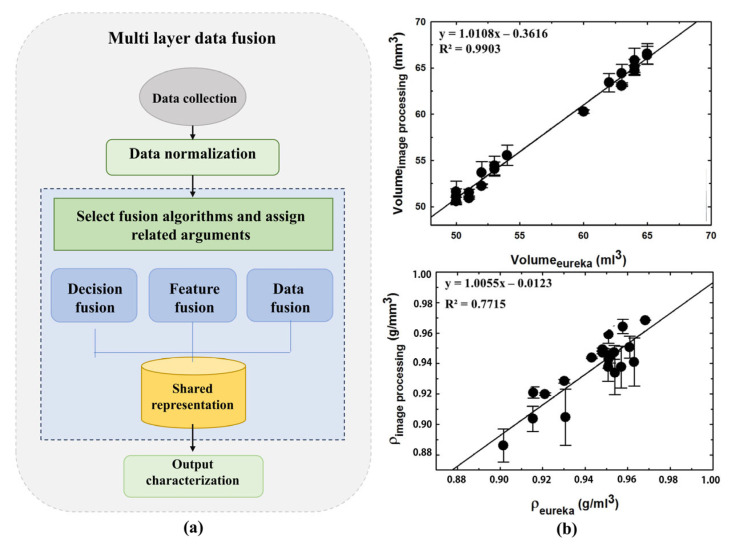
Multi-sensor fusion strategies and machine vision characterization of egg quality: (**a**) The general process of multi-sensor fusion. (**b**) Egg volume and density were measured by a machine vision system [87].

As illustrated in Figure 5a, based on the hierarchy of information processing, multi-sensor fusion encompasses three distinct paradigms: first, data-level (low-level) fusion, which directly concatenates and analyzes raw data from multiple heterogeneous sources. This strategy usually requires large-scale models and massive datasets for effective training; second, feature-level (mid-level) fusion, which extracts representative features from individual sensing datasets prior to integration. While this reduces the dimensionality of the input data, it may result in the loss of fine-grained cross-modal information. Third, decision-level (high-level) fusion, which generates independent predictions from each sensor and subsequently combines the outputs for a unified decision. This offers high computational efficiency, but it cannot capture dependencies between different modalities at the feature level [81,82]. These three hierarchical fusion strategies correspond to successive stages of information processing, offering flexible and tailored solutions for diverse detection scenarios. For instance, feature-level fusion is recommended for integrating datasets from divergent spectroscopic platforms or combining spectral and non-spectral sensing data; data-level or feature-level fusion is applicable for image-spectroscopy fusion; and decision-level fusion is only preferable when the sole output is a definitive classification or grading decision [83]. When performing fusion, it is important to be aware of potential practical issues, such as missing modal data or spatiotemporal misalignment of data from multiple sources, which can limit its applicability [84]. Benefiting from its inherent merits—including reduced measurement error, enhanced anti-interference capacity, and extended sensing dimensionality—multi-sensor fusion has emerged as a pivotal driver advancing technological innovation in non-destructive egg quality evaluation.

While each of the aforementioned standalone spectroscopic techniques exhibits unique merits, single-modal spectroscopy is susceptible to matrix interferences, environmental fluctuations, and other inherent constraints, which limit detection robustness and the comprehensiveness of acquired quality-related information. Accumulating evidence validates that such multi-modal fusion strategies effectively circumvent the bottlenecks of single-technology detection: the synergistic integration of HSI systems and electronic noses (e-noses) allows simultaneous acquisition of spectral signatures and volatile organic compound (VOC) profiles of eggs. Specifically, Zhang et al. developed a Multi-Data Fusion Attention Network (MDFA-Net) by fusing e-nose and hyperspectral data, attaining a classification accuracy of 99.88% for egg freshness evaluation [85]. Similarly, Çiftçi et al. constructed a robust egg storage time prediction model by integrating UV–Vis spectroscopy, fluorescence spectroscopy, and physicochemical parameters (pH and color), with a $R_{P}^{2}$ of 0.97 [86].

In industrial and on-site applications, the cross-dimensional integration of complementary physical sensors offers substantial practical advantages. The combination of machine vision (for external dimensional measurements) and load-cell weighing sensors enables non-destructive determination of egg volume and density with accuracies exceeding 99%, as presented in Figure 5b. The coefficient of determination (R^2^) between egg density and freshness reached 0.9982, indicating an extremely strong linear correlation. This cost-effective and easily implementable strategy is highly compatible with the practical requirements of small- and medium-sized egg processing enterprises [87].

It is noteworthy that the effectiveness of multi-sensor fusion does not increase proportionally with the number of sensors; rather, it hinges on mutual correction and complementarity among different sensors. Thus, the combination of near-infrared transmission spectroscopy with low-resolution proton nuclear magnetic resonance (LRH NMR) technology does not yield superior results compared to their individual applications [88]. Evidently, fusion does not always result in performance gains. In addition, more sensors also mean greater risks. A failure in a single sensor can propagate through the fusion link, and the probability of failure is higher in multi-sensor systems [89]. Therefore, sensors should be selected based on a scientific analysis of modal correlations to ensure that complementary modalities correspond to distinct yet related layers of quality information, thereby improving detection accuracy and stability [90]. Collectively, multi-sensor fusion technology exhibits distinct advantages in enhancing the accuracy, stability, and reliability of egg freshness detection models. However, the widespread industrial translation of this technology remains hindered by several critical challenges, including high instrumentation costs, poor interpretability of complex fusion algorithms, and substantial technical barriers to sensor miniaturization. Future research should focus on finding the optimal balance between interpretability and model performance, utilizing metrics such as global feature importance and permutation importance to quantify the role of features and assess the contributions of modal and feature levels, in order to determine whether the model truly benefits from complementary information or is merely dominated by a single high-dimensional modality [91]. At the same time, it is also important to optimize hardware systems to reduce deployment costs, thereby promoting the sustainable and practical development of this technology.

## 4. Data Analysis Combined with AI Technology

**Figure 6:**
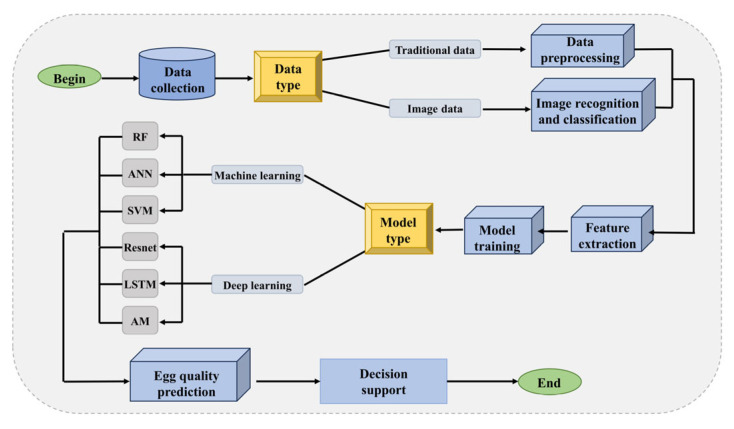
Application process of artificial intelligence in egg quality detection. Note: The arrows indicate the direction of data transmission.

Artificial Intelligence is a branch of computer science that enables machines to mimic human intelligent behaviors via programs and algorithms, with core capacities of data learning, reasoning, and decision-making. As the foundational enabling technology of AI, machine learning empowers machines to conduct adaptive learning from data [92]. Figure 6 exhibits the hierarchical framework of AI for egg freshness detection, including bottom-level ML modeling, middle-level deep learning optimization, and top-level interpretable portable detection, covering algorithm selection, model optimization, and practical translation. Unlike classical chemometrics, which is primarily used for spectral preprocessing, dimensionality reduction, and linear modeling, machine learning enables autonomous learning, feature extraction, and improved accuracy in spectral detection models. Consequently, its integration with spectroscopy has become a core research direction in this field. The widely used ML models cover decision trees (DT), random forests (RF) [93], support vector machines (SVM) and artificial neural networks (ANN) [94], and related studies have verified their excellent performance: structured ANNs and BNs achieve 100% classification accuracy, while the stepwise regression-cuckoo search-support vector machine (SWR-CS-SVM) model reaches 96% accuracy, making ML an essential tool for egg quality detection [95].

As a key component of artificial intelligence, machine learning extracts useful information from data and enables autonomous model learning relying on manually pre-extracted features [96]. Its introduction can significantly enhance the accuracy of spectral-based quality model predictions and classification. Currently, the fusion of machine learning and spectroscopy has become a core development direction in the field of egg freshness detection. Researchers continue to explore the application effects of different machine learning algorithms in egg freshness detection, aiming to screen out the model with the optimal accuracy. A study conducted by Sun et al. utilized hyperspectral imaging technology. Through comparative analysis of multiple algorithms, the SWR-CS-SVM model emerged as the most effective, achieving a classification accuracy of 96% [97]. These studies demonstrate that the integration of spectroscopy technology and machine learning can stably achieve high detection accuracy, thereby providing a reliable method for the efficient evaluation of egg freshness.

**Figure 7:**
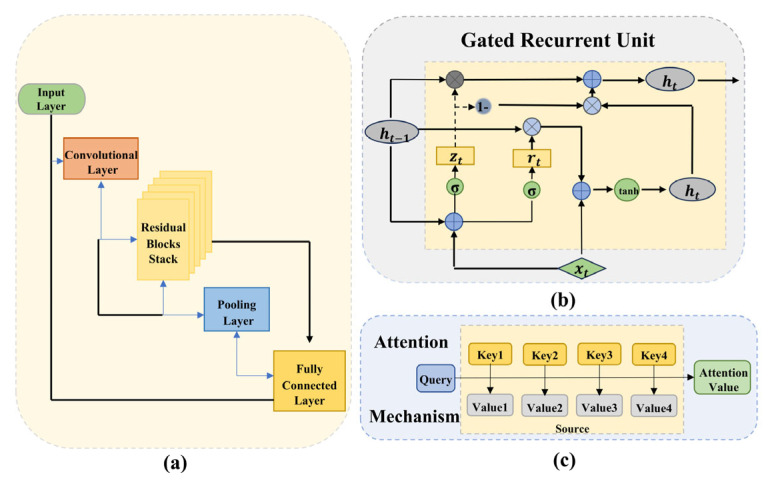
Schematic of core deep learning modules: (**a**) Schematic diagram of ResNet. (**b**) Schematic diagram of Gated Recurrent Unit (GRU). (**c**) Schematic diagram of the attention mechanism. Note: In Figure 7a, the black arrows represent the forward propagation flow of feature data; the light blue arrows represent the residual shortcuts in ResNet. In Figure 7b, the solid arrows indicate the main direction of forward propagation of feature data, while the dashed arrows represent the computational branches for updating the complementary coefficients of the gates. In Figure 7c, the horizontal arrows indicate attention weights, and the vertical arrows represent correspondences.

As a core advanced technology under the AI framework, deep learning significantly outperforms traditional algorithms in processing unstructured image and spectral data by virtue of the robust feature learning and nonlinear fitting capabilities of deep neural networks [98]. Convolutional neural networks (CNNs), with convolutional layers at their core, progressively filter out irrelevant and redundant information layer by layer, demonstrating excellent local representation capabilities [99]. Ong et al. constructed a one-dimensional convolutional neural network (1D-CNN) model using hyperspectral imagery and compared it with algorithms such as PLSR, SVR, and gradient-boosted trees. The results show that the convolutional neural network model achieved the highest correlation coefficient and the lowest error on both the validation and test sets [48]. The newly proposed continuous wavelet transform-convolutional neural network (CWT-CNN) model, which uses CWT processing to enhance the feature information in spectral data, has achieved a $R_{P}^{2}$ of 0.9059 for egg freshness prediction and a recognition accuracy of 90.7% for egg freshness grades [100]. However, CNNs have limitations due to their weak capability in modeling temporal dependencies. In contrast, recurrent neural networks (RNNs) perform well in processing sequential data but struggle to model spatial features. Moreover, both networks suffer from the gradient vanishing problem in practical applications. As task complexity continues to increase, the demand for more advanced optimization algorithms becomes increasingly prominent. ResNet—an advanced variant of convolutional neural networks—has been developed. As shown in Figure 7a, it adopts an architecture consisting of an “input layer—convolution layer—stacked residual blocks—pooling layer—fully connected layer.” By introducing residual connections, information can be directly passed between different layers, enabling the network to be easily stacked into structures with dozens or even hundreds of layers, thereby effectively addressing the challenges associated with training deep networks [101]. The emergence of RNN variants, such as LSTM and GRU, has also addressed the limitations of traditional RNNs [102]. Figure 7b illustrates the architecture of the Gated Recurrent Unit (GRU), a lightweight LSTM variant The unit processes the current input $x_{t}$ and previous hidden state $h_{t-1}$ via Sigmoid (σ)-activated update $z_{t}$ and reset $r_{t}$ gates for temporal information fusion, outputs the current hidden state $h_{t}$, and resolves the vanishing gradients and long-range dependency issues of traditional RNNs. Other studies have demonstrated that by combining CNNs and RNNs, it is possible to leverage both spatial structural information and temporal patterns to achieve complementary advantages [103]. Previous research has shown that combining convolutional neural networks with a variant of recurrent neural networks—the Long Short-Term Memory (LSTM) algorithm—and applying transfer learning techniques can enhance data adaptability and yield superior predictive performance [104]. As a supplementary module for deep learning, the introduction of the attention mechanism (AM) allows the use of limited computing resources to process more critical information in scenarios with limited computing power [105]. AM centers on QKV matching; it assigns weights by calculating the similarity between queries (Query) and keys (Key), and then performs a weighted sum of the values (Value), thereby achieving a focus on key features. Figure 7c illustrates this concept. The specific deep learning architecture should be selected based on the nature of the input data and the prediction task. For example, CNNs excel at extracting local spatial and spectral features and are suitable for hyperspectral and near-infrared data, as well as tasks dominated by local patterns, while LSTMs are better suited for modeling spectral data in sequences [106].

Table 2 summarizes the various AI-assisted technologies applied for egg freshness detection. Although AI models have demonstrated high performance in the field of quality inspection, their application also faces many challenges. First, AI models rely on vast amounts of high-quality data for training and prediction, which is not easily obtained in the food industry. Therefore, it is essential to prioritize data collection, annotation, and storage to ensure that the datasets are reliable and representative [107]. Second, AI models can be complex and difficult to interpret, and the growing prominence of the “black box problem” has led to an increasing demand for explainable artificial intelligence (XAI) technology [108]. In recent research in this field, Ahmed et al. demonstrated that combining near-infrared spectroscopy with explainable artificial intelligence can provide mechanistic explanations for the predictive processes of spectral AI models, thereby enhancing the reliability of detection results [109]. For future applications, priority should be given to using traditional machine learning models or simplified deep learning architectures to build “intrinsically explainable” models [109]. If the task complexity is high and deep learning must be adopted, post hoc interpretation techniques can be used to externally explain the model, thereby better meeting the trust requirements in practical scenarios [110]. In line with OECD principles for trustworthy machine learning, spectral AI models need standardized validation. Researchers should adopt Y-randomization and permutation tests for robustness verification, conduct uncertainty quantification, define clear applicability domains, and report performance across multiple independent biological batches to ensure reliable and credible model deployment [111]. To accelerate the commercialization of spectral AI models, efforts should be made to integrate portable devices with advanced deep learning technologies, thereby further enhancing detection capabilities and enabling real-time online detection [112]. At the same time, internet of things (IoT) technology should be leveraged to overcome temporal and spatial constraints in detection [107], and a cloud-based AI analysis platform should be established to enable real-time, remote, and dynamic monitoring. These research directions are expected to drive transformative upgrades in the egg industry and hold significant research value for further in-depth exploration.

## 5. Conclusions

Hen egg quality assessment is of great significance for ensuring food safety and commercial value. As non-destructive tools for signal acquisition in egg freshness detection, spectroscopic techniques have shown great application potential. Visible-near-infrared spectroscopy stands as the most mature and widely adopted solution due to its low cost, fast detection speed and excellent portability, which makes it well-suited for industrial online inspection. In contrast, hyperspectral imaging, Raman spectroscopy and fluorescence spectroscopy deliver superior performance in detection accuracy and molecular characterization, yet their practical deployment is largely constrained by hardware limitations. To address the drawbacks of single-mode detection, multi-sensor fusion has been introduced to integrate data and features from various spectroscopic devices and auxiliary sensors, effectively improving the overall stability of detection systems.

In terms of modeling, conventional machine learning, which constructs prediction and classification models based on manually extracted spectral features, remains the dominant approach for spectral data analysis. In recent years, increasing attention has been paid to deep learning represented by convolutional neural networks (CNNs) and long short-term memory (LSTM). As key branches of artificial intelligence, these architectures can automatically extract in-depth features and fit complex nonlinear relationships, thereby substantially improving the modeling accuracy for high-dimensional spectral and image data. Nevertheless, the inherent black-box issue of deep learning models hinders their large-scale industrial implementation. In this context, XAI serves as a post hoc interpretation tool to reveal the intrinsic correlations between spectral signatures and biochemical changes in eggs and further enhance model transparency and reliability.

Currently, high hardware costs, environmental interference and poor model generalization across distinct biological batches are the primary obstacles to the real-world application of spectral intelligent detection systems. Additional challenges include dataset imbalance, inadequate external validation and difficult calibration transfer. The field also lacks unified benchmark datasets for fair algorithm comparison. Meanwhile, standardized validation procedures including Y-randomization, permutation tests, uncertainty quantification and applicability domain definition need to be established in accordance with the OECD principles for trustworthy machine learning. Future research should focus on developing miniaturized, low-cost portable spectroscopic devices and constructing multi-sensor fusion frameworks with stronger complementarity and stability, while promoting the practical application of XAI. The integration of these technologies into a complete system will enable real-time monitoring across the entire egg supply chain from farm to table.

## Figures and Tables

**Table 1:** Comparison of advanced techniques in egg freshness detection.

Spectroscopic Technique	Vis-NIR Spectroscopy	Hyperspectral Imaging	Raman Spectroscopy	Fluorescence Spectroscopy
Cost	Low	High	Moderate	Moderate
Sample throughput	High	Relatively low	Moderate	Moderate
Eggshell interference robustness	Moderate	Relatively high	Relatively low	Relatively low
Portability	High	Relatively low	Moderate	Moderate
Need for preprocessing	Relatively low	High	Relatively high	Relatively high

**Table 2:** Summaries of different AI-assisted technologies applied for egg freshness detection.

Category	Detection Indicators	Model	Performance	References
Vis-NIR spectroscopy	Albumen pH	PLSR	$R_{P}^{2}$ = 0.824, RMSEP = 0.133	[19]
NIR spectroscopy	Haugh unit, yolk index, and weight loss.	Si-PLS	$R_{P}^{2}$ = 0.666, 0.824, 0.769; RMSEP = 4.250, 0.031, and 0.005, respectively.	[18]
NIR spectroscopy	Haugh unit	PLS-R and SVM-R	Accuracy of 87%	[32]
NIR spectroscopy	Haugh unit	CWT-CNN	Accuracy of 90.7%	[100]
NIR spectroscopy	Weight, major diameter, and shell thickness	PLSR, RF, GBM, and KNN	RMSEP = 1.42 (optimum)	[93]
Hyperspectral imaging	Haugh unit	CNN	Accuracy of 99%	[49]
Hyperspectral imaging	Haugh unit	DC-CNN	$R_{P}^{2}$ = 0.820, RMSEP = 4.415	[48]
Micro-Raman spectroscopy	Total weight, yolk weight, egg shape index, yolk index, and yolk coefficient	PLS-DA	Accuracy of 80%	[60]
Raman spectroscopy	Haugh unit, albumen pH, and air chamber diameter	PLSR	$R_{P}^{2}$ = 0.712 and RMSEP = 2.933, $R_{P}^{2}$ = 0.810 and RMSEP = 0.047, $R_{P}^{2}$ = 0.707 and RMSEP2.380, respectively.	[56]
Synchronous Fluorescence spectra	Haugh unit	MLR	$R_{P}^{2}$ = 0.888, SEP = 6.290	[74]
Dielectric spectroscopy	Air chamber diameter	ANN, BN, DT, SVM	Accuracy of 100%, 100%, 87.5%, 100%, respectively	[95]
Hyperspectral	The origin of table eggs	SVM	Accuracy of 96%	[104]
Transfer Learning	Haugh unit and weight	CNN-LSTM	$R_{P}^{2}$ = 0.888, RMSEP = 1.650	[88]
Hyperspectral imaging	Texture features, characteristic images, Haugh unit	KNN, RF, PLS-DA, SVM and MLP	The highest Accuracy of 92.4% (SG-FD-SNV)	[35]

Notes: Si-PLS, Synergy Interval Partial Least Squares; SVM-R, Support Vector Machine-Regression; GBM, Gradient Boosting Machine; DT, Decision Tree; BN, Batch Normalization; MLP, Multilayer Perceptron; $R_{p}^{2}$, Coefficient of Determination of the Prediction Set; RMSEP, Root Mean Square Error of Prediction; SEP, Standard Error of Prediction.

## Data Availability

No new data were created or analyzed in this study.
